# Posttransplant lymphoproliferative disorder after uterus transplantation

**DOI:** 10.3389/ti.2026.16946

**Published:** 2026-08-20

**Authors:** Liza Johannesson, Kimia Samiee, Luis Pineiro, Cedric W. Spak, Metin Punar, Giuliano Testa

**Affiliations:** 1 Annette C. and Harold C. Simmons Transplant Institute, Baylor University Medical Center, Dallas, TX, United States; 2 Department of Obstetrics and Gynecology, Baylor University Medical Center, Dallas, TX, United States; 3 Texas A&M University, Dallas, TX, United States; 4 Department of Oncology, Baylor University Medical Center, Dallas, TX, United States; 5 Department of Infectious Disease, Baylor University Medical Center, Dallas, TX, United States; 6 Department of Pathology, Baylor University Medical Center, Dallas, TX, United States

**Keywords:** infection, infertility, PTLD, recipient, uterus transplant

Dear Editors,

Posttransplant lymphoproliferative disorder (PTLD) comprises a spectrum of abnormal lymphoid proliferations that occur in the setting of posttransplant immunosuppression [1]. Epstein–Barr virus (EBV) plays a central role in most cases [2], as impaired T-cell immune surveillance permits proliferation of EBV-infected B cells, ranging from polyclonal expansion to malignant lymphoma.

Although PTLD is well described following solid-organ and hematopoietic transplantation, its occurrence after vascularized composite allografts (VCAs), particularly uterus transplantation (UTx), remains poorly characterized [3]. As UTx becomes an established treatment for absolute uterine-factor infertility, reporting rare but serious complications is essential for informing patient counseling, risk assessment, and immunosuppressive management.

We experienced a case of PTLD that developed during the postpartum period following UTx. A 38-year-old woman with absolute uterine-factor infertility due to Mayer-Rokitansky-Küster-Hauser syndrome underwent living-donor UTx in April 2023 (Figure 1). Her medical history included chronic hypertension and a body mass index of 26 kg/m^2^. Pretransplant testing showed negative EBV viral capsid antigen IgG and IgM, indicating no detectable EBV antibodies, while cytomegalovirus (CMV) IgG was positive. *In vitro* fertilization with cryopreservation of euploid embryos was completed before transplantation.

**FIGURE 1:**
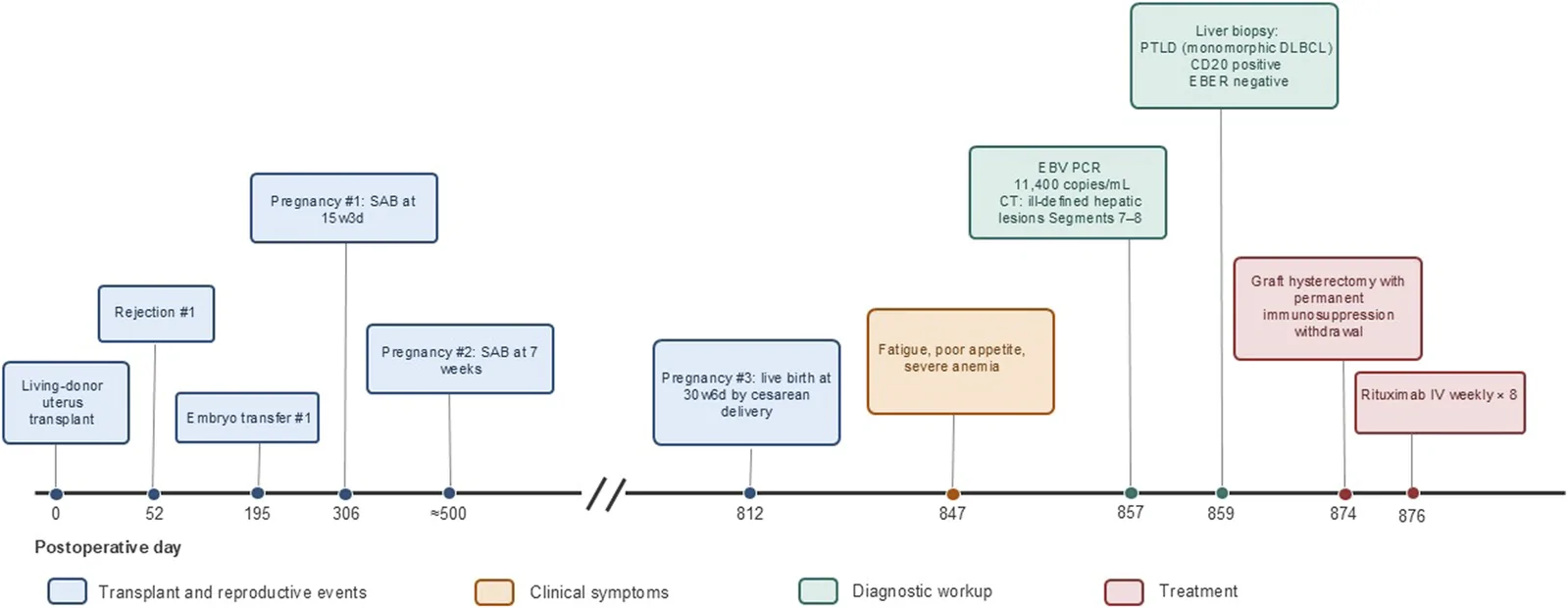
Clinical timeline of uterus transplantation and posttransplant lymphoproliferative disorder.

The donor was a 40-year-old parous woman who was EBV IgG-positive and CMV IgG-negative. Cold and warm ischemia times were 151 and 87 min, respectively, and the surgery was uncomplicated.

Induction immunosuppression consisted of antithymocyte globulin (total 4.5 mg/kg), followed by maintenance therapy with tacrolimus, azathioprine, and prednisone. Tacrolimus targets were later reduced. CMV and Pneumocystis prophylaxis were administered for 3 months, and fluconazole was given for 1 month.

Menses resumed on posttransplant day (PTD) 32. One episode of moderate T-cell–mediated rejection on PTD 52 resolved with intravenous steroids. No infections were documented after transplantation. Embryo transfer began on PTD 249. Four transfers resulted in three clinical pregnancies and one implantation failure. Two pregnancies ended in spontaneous abortion, including one at 15.3 weeks associated with placenta previa and bleeding requiring dilation and curettage.

The third pregnancy resulted in a preterm live birth. Cervical insufficiency required cerclage at 21 weeks. At 30 weeks and 6 days, premature rupture of membranes and preterm labor occurred, leading to cesarean delivery of a healthy male infant weighing 1.46 kg. The infant’s neonatal intensive care unit course was uncomplicated.

On postpartum day (PPD) 35 (PTD 847), the patient reported progressive fatigue and poor appetite. Physical examination demonstrated pallor and an inflamed cervix. Laboratory studies revealed anemia with a hemoglobin level of 7.9 g/dL. Cervical biopsy demonstrated severe T-cell–mediated rejection, and high-dose intravenous steroids were administered.

Despite treatment, she developed worsening constitutional and gastrointestinal symptoms accompanied by a 4 kg weight loss. Persistent anemia and leukopenia were present, and EBV polymerase chain reaction testing demonstrated a viral load of 11,400 copies/mL.

Computed tomography of the chest and abdomen identified new hypodense lesions in hepatic segments 7 and 8, the largest measuring 4.4 × 4.0 cm, without thoracic involvement. Lactate dehydrogenase was elevated. Given the combination of EBV viremia and hepatic lesions, immunosuppression was discontinued. Histopathology of a liver biopsy demonstrated PTLD classified as diffuse large B-cell lymphoma, positive for CD20 and negative for EBV-encoded RNA (EBER) (Figure 2). Withdrawal of immunosuppression led to declining EBV viral loads.

**FIGURE 2:**
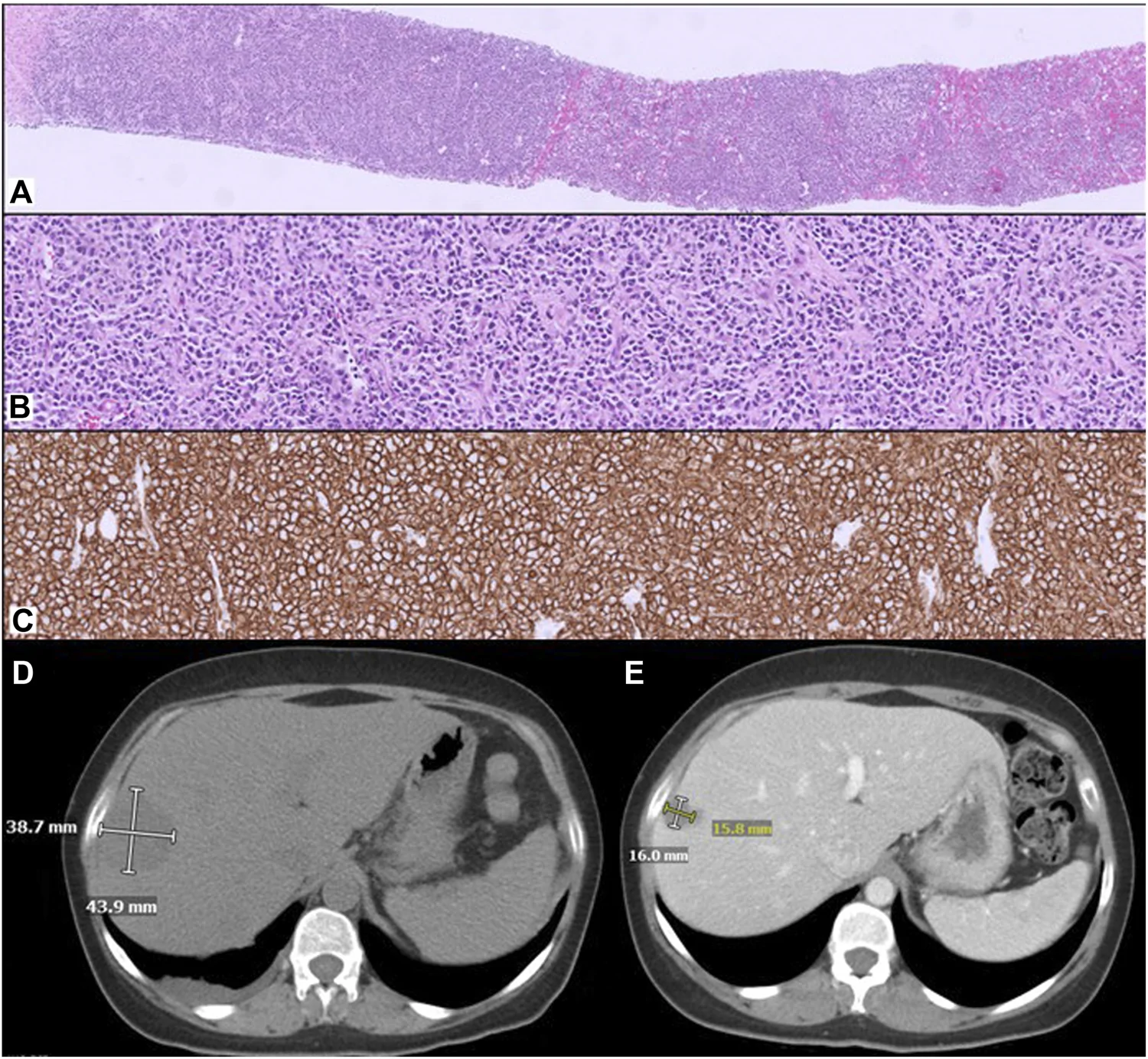
**(A)** Hematoxylin and eosin (H&E), ×50 magnification showing neoplastic cells infiltrating through hepatic parenchyma in a diffuse sheet-like growth pattern. **(B)** H&E, ×400 magnification showing large lymphocytes with nuclear hyperchromasia, atypia, nuclear membrane irregularity, and amphophilic to basophilic cytoplasm. **(C)** CD20 immunohistochemistry, ×400 magnification showing diffuse positivity for CD20. Computed tomography of the chest and abdomen showing **(D)** hepatic lesions, the largest in segment 8 measuring 4.4 × 3.9 cm on posttransplant day 857 and **(E)** decreased size of the hepatic lesions, suggesting a positive treatment response following 8 doses of rituximab.

Thirty days after symptom onset, the patient was admitted, and on PPD 62 (PTD 874), graft hysterectomy was performed to permit permanent discontinuation of immunosuppression.

Postoperatively, intermittent fevers secondary to urinary tract infection caused by *Klebsiella* and *Escherichia coli* were successfully treated with antibiotics. Rituximab (375 mg/m^2^ weekly) was initiated for four planned cycles. Follow-up imaging 51 days later demonstrated partial response with reduction in hepatic lesion size, prompting four additional weekly cycles. At 3-month follow-up after hysterectomy, the patient reported substantial clinical improvement. Imaging demonstrated continued regression of hepatic lesions without new disease, positron emission tomography showed no metabolic activity, and EBV viral load was undetectable. At 12-month follow-up after hysterectomy PET scan showed no evidence of fluorodeoxyglucose (FDG) avid lymphoma.

PTLD is a recognized complication of immunosuppression, most commonly resulting from impaired T-cell surveillance and uncontrolled B-cell proliferation [2, 4]. Although extensively reported after kidney and other solid-organ transplantation, PTLD is exceedingly rare in VCAs [2, 5–7]. Only four previous cases have been reported among nonuterus VCA recipients. These cases included recipients of hand, lower-extremity, facial, and upper-extremity transplants. Presentations ranged from EBV-associated diffuse large B-cell lymphoma to late-onset EBV-negative PTLD. Management strategies included reduction or withdrawal of immunosuppression, rituximab-based therapy, and, in some cases, graft explantation, with variable outcomes [2, 5–7].

EBV mismatch between donor and recipient is a well-established risk factor for EBV-positive PTLD in solid-organ transplantation, and routine EBV monitoring is standard practice [2, 4]. However, the relationship between EBV mismatch and EBV-negative PTLD remains less clear. Because UTx is intended to restore fertility rather than preserve life, the balance between benefit and long-term risks differs fundamentally from that of traditional organ transplantation.

In this patient, EBV viremia prompted further evaluation, yet histopathology demonstrated EBER-negative diffuse large B-cell lymphoma. This finding highlights that PTLD may develop independently of direct EBV-driven oncogenesis, particularly after prolonged or intensive immunosuppression [2, 4]. EBV-negative PTLD is thought to arise through alternative mechanisms, including accumulation of genetic alterations and impaired immune surveillance related to chronic immunosuppression. Importantly, EBV DNAemia does not necessarily indicate that the lymphoma is EBV-driven. The patient’s cumulative exposure to thymoglobulin induction, triple-agent maintenance therapy, and repeated high-dose steroids for rejection likely contributed to PTLD development.

Donor-recipient EBV mismatch deserves particular consideration in UTx. Although donor selection is often constrained by anatomical and logistical factors, the elective nature of UTx raises questions regarding acceptable infectious and oncologic risks. Whether EBV mismatch should influence candidate selection, surveillance protocols, or immunosuppressive strategies remains uncertain and warrants further study.

Management of PTLD in UTx differs from that in life-sustaining organ transplantation. Because the uterus is a temporary graft, hysterectomy permits permanent discontinuation of immunosuppression. In this case, graft removal combined with rituximab therapy resulted in favorable clinical and radiographic outcomes.

To our knowledge, PTLD following UTx has previously been mentioned only in aggregate reporting without detailed clinical characterization [1]. This case provides a comprehensive description of presentation, diagnostic findings, management, and short-term outcomes. This case highlights the importance of EBV risk assessment, careful immunosuppression management, and vigilant surveillance in UTx. Continued reporting of rare complications will be critical to refining patient selection, counseling, and long-term management strategies in this evolving field.

## Data Availability

The datasets presented in this article are not readily available because Single patient data; data will not be available due to patient confidentiality. Requests to access the datasets should be directed to liza.johannesson@bswhealth.org.
